# Conformational Rearrangement of Fission DSPs

**DOI:** 10.1002/bies.70062

**Published:** 2025-09-01

**Authors:** Anelise N. Hutson, Kristy Rochon, Jason A. Mears

**Affiliations:** ^1^ Department of Pharmacology Case Western Reserve University School of Medicine Cleveland Ohio USA; ^2^ Department of Biological Sciences Purdue University West Lafayette Indiana USA; ^3^ Cleveland Center for Membrane and Structural Biology Case Western Reserve University School of Medicine Cleveland Ohio USA; ^4^ Center for Mitochondrial Research and Therapeutics Case Western Reserve University School of Medicine Cleveland Ohio USA

## Abstract

Dynamin superfamily proteins (DSPs) are large GTPases that play crucial roles in membrane remodeling processes, including vesicle uptake, mitochondrial fission, and opposing fusion events. Among them, dynamin and dynamin‐related protein 1 (Drp1) share a conserved domain architecture, yet exhibit unique structural and regulatory features that tailor their functions. This review explores the conformational rearrangements of the mammalian fission DSPs, dynamin and Drp1, focusing on their dimeric and tetrameric structures, lipid‐bound assemblies, and key regulatory elements that drive membrane constriction. Structural biology methods, including x‐ray crystallography and cryo‐electron microscopy, have provided insight into the mechanism of activation and constriction of these DSPs, revealing how domain interactions and intrinsically disordered regions regulate self‐assembly and enzymatic activity. We briefly examine the role of sequence modifications and partner proteins in modulating DSP function, highlighting the impact of regulatory factors on their respective cellular functions. An ongoing goal is to better understand the molecular mechanisms governing the transitions from a pre‐assembled cytosolic state to a self‐assembled state for dynamin and Drp1 on membranes, which provides a foundation for studying subsequent helical constriction. This insight will enhance our knowledge of organelle dynamics and provide new avenues for therapeutic interventions targeting DSP‐related pathologies.

## Graphical abstract

Assembly properties of fission DSPs. Fission DSPs exist in a dynamic equilibrium of oligomer states in the cytosol, where they must be activated by partner proteins or lipid to induce assembly, and upon GTP hydrolysis, constriction.

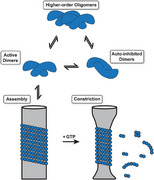

## 1 Introduction

Dynamin superfamily proteins, or DSPs, are a family of large GTPases involved in membrane remodeling events. The founding member of this superfamily, dynamin, was discovered initially in rat brains [1], and later was connected with the temperature‐sensitive paralysis caused by the *shibire* mutation in *Drosophila melanogaster* [2]. Dynamins form collars around the necks of budding vesicles during endocytosis, and the *shibire* mutation in dynamin prevented vesicle uptake at neuronal synapses [3]. Similarly, DSPs are generally lipid‐interacting proteins that regulate a variety of cellular membrane remodeling events through coordinated GTPase activity. These events include vesicle uptake [4], mitochondrial fission [5] and fusion [6], viral protection [7], cell division [8], and other membrane dynamics processes.

In the past few decades, research into the role of DSPs has highlighted their importance in maintaining cellular health, and their compromised function has been associated with cancers [9, 10, 11, 12], neurodegenerative diseases [13, 14, 15, 16], vision loss [17], heart disease [18], and viral infections [19]. Though the domain architecture of DSPs is generally conserved, differences define specific functions in cellular activities. Therefore, structural studies of DSPs provide insight into their core properties and delineate key regulatory elements that are often disrupted in human disease. In this review, we describe structural features of dynamins and dynamin‐related protein 1 (Drp1) as it relates to the constriction of lipid membranes. Specifically, we will examine their multimer structures in solution, larger assemblies around lipid templates, the location and impact of loops and disordered regions in these structures, and conserved regulatory components in DSPs.

## 2 The Domain Architecture of DSPs

**FIGURE 1:**
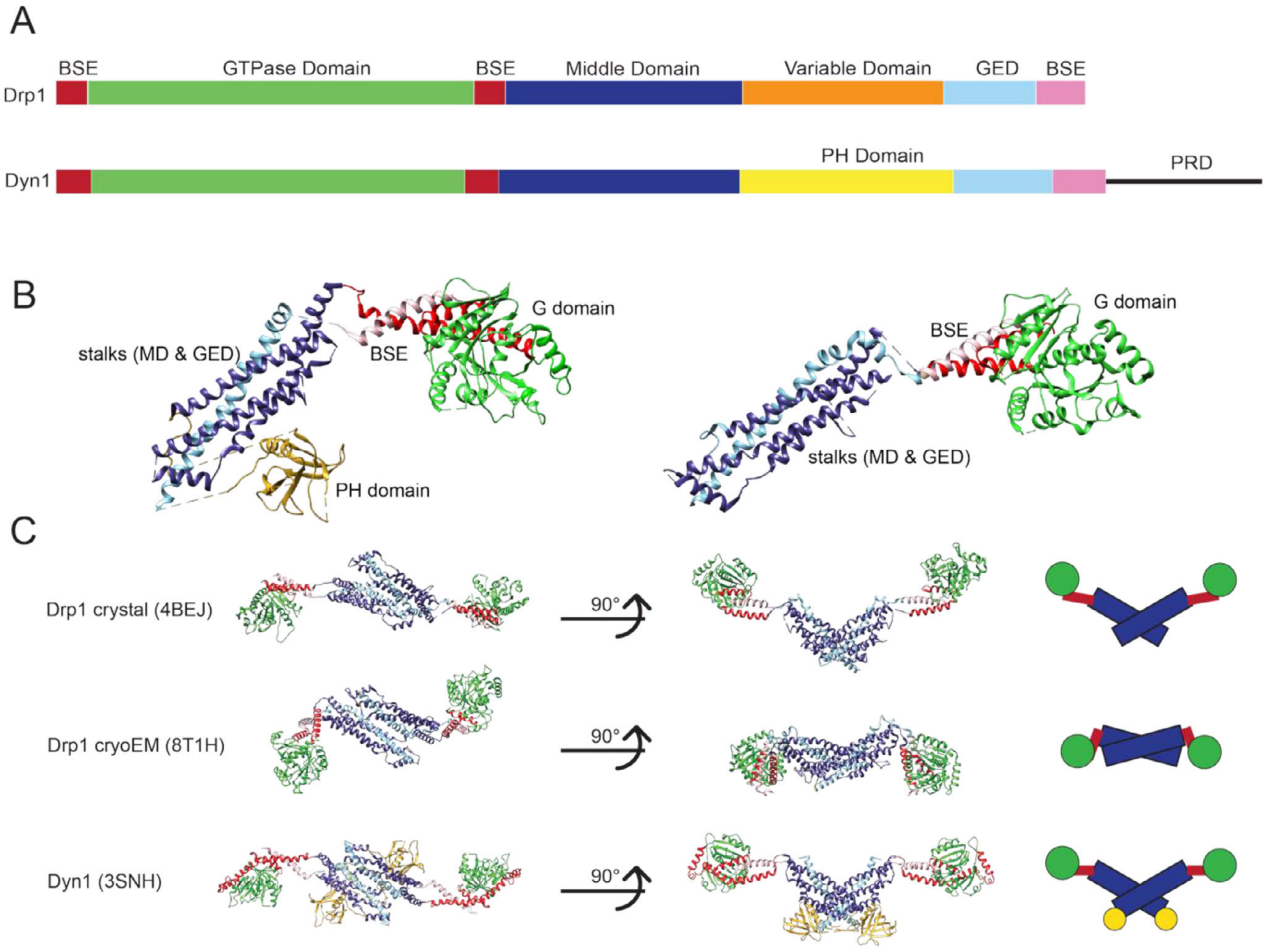
Dynamin superfamily protein (DSP) fission protein structures. (A) Structure‐based sequence visualization of dynamin‐related protein 1 (Drp1) and a dynamin, Dyn1. (B) Monomers of Dyn1, pictured left, and Drp1, pictured right. DSP fission proteins have three common domains: GTPase domain, middle domain, and GTPase effector domain (GED), and a bundling signaling element (BSE). Drp1 and dynamins also have functional inserts unique to their sequences: Drp1 has a disordered variable domain (VD, not shown), and dynamin has a pleckstrin homology (PH) domain and a disordered proline‐rich domain (PRD, not shown). (C) DSP fission protein structures have been resolved in non‐assembled dimers. Drp1 dimer structures have been published from a crystal structure (PDB ID: 4BEJ) and from a cryoEM density (PDB ID: 8T1H). Dyn1 was published from a crystal structure (PDB ID: 3SNH).

DSPs have three characteristic domains as shown in Figure 1: the GTPase (G) domain, responsible for enzymatic activity, the middle domain (MD), which forms interactions necessary for oligomerization, and the GTPase effector domain (GED), which also contributes to inter‐ and intramolecular domain interactions [20]. The MD and GED together form the stalk, which contains sites for conserved intermolecular interfaces important for self‐assembly. The extensive variation in domains within DSPs has been covered in other reviews [21, 22, 23, 24, 25]. Here, we focus exclusively on mammalian fission proteins: Drp1 and dynamins, of which there are three genes encoding dynamin‐1, dynamin‐2, and dynamin‐3. Dynamin‐1 is primarily expressed in the brain [26], dynamin‐3 in the brain and testis [27, 28], and dynamin‐2 and Drp1 are expressed more ubiquitously [29, 30]. Dynamins are responsible for endocytosis and vesicle trafficking, with dynamin‐1 and dynamin‐3 playing important roles in clathrin‐mediated endocytosis at the synaptic cleft [31]. Drp1 is the major regulator of mitochondrial fission [32]. All of these proteins are cytosolic and share key domain organization features.

The first ∼20 amino acids in fission DSPs contribute to an additional domain known as the bundle signaling element (BSE) [33]. Specifically, these initial residues at the N‐terminal end of the G domain form a helix that interacts with two additional helices to form a three‐helix bundle. The second helix is at the C‐terminal end of the G domain, and the third is contributed by a C‐terminal portion of the DSP protein sequence, hundreds of residues from the first two and proximal to the aforementioned GED. With this topology, the BSE is able to confer conformational changes associated with GTP hydrolysis into a powerstroke motion in the stalk that results in altered intermolecular interactions that mediate constriction [34].

At the opposing end of the 3D protein structure, membrane fission proteins have conserved lipid‐binding domains that target membrane interactions. Drp1 contains a variable domain (VD) region that facilitates mitochondrial membrane interactions, and it has also been shown to regulate self‐assembly through auto‐inhibitory interactions [35, 36, 37]. Recently, Drp1 was found to have a smaller C‐terminal Short Linear Motif, called the CT‐SLiM, that allosterically regulates its structure and function [38]. In the same position as the VD, dynamins contain a pleckstrin homology (PH) domain that senses membrane curvature and regulates assembly diameter [39]. Dynamins’ sequences and domain structures are largely homologous with the exception of the disordered proline‐rich domain (PRD), an ∼100 amino acid sequence at the C‐terminal end of dynamin. Differences in the PRD sequence between the three dynamin proteins likely distinguish functional interactions with binding partners, including SH3‐containing proteins [40], and regulation of disassembly from the membrane [41].

DSPs are part of the translation factor‐related (TRAFAC) class of GTPases due to their phosphate‐binding loop, or P loop, sharing the conserved GXXXXGK sequence [42]. However, they are unique in their low affinity for nucleotide, and their “molecular switch” does not transmit a signal, but rather induces self‐assembly and stimulates enzymatic activity [43]. The P‐loop uses a hydrogen‐bonded network to coordinate the binding of the phosphate group on the nucleotide, and a universally conserved lysine residue (Position 38 in Drp1 and 44 in dynamins) interacts with the phosphate backbone. Mutation of this lysine to an alanine results in an enzymatically impaired protein [44, 45]. Unlike other GTPases, the relative low affinity for nucleotide binding in DSPs permits nucleotide exchange without the need for additional cofactors to complete GTP hydrolysis cycle.

The first molecular insight into DSPs came from structural studies of the isolated PH domain of dynamin‐1 [46]. Following this advance, crystallography methods were able to resolve truncated GTPase domains and stalk structures of DSP constructs, providing insight into distinct domains [47, 48]. A major breakthrough came in 2011 when the structure of dynamin‐1 was reported from the Daumke and Nunnari labs [49, 50]. These refined studies demonstrated the topological relationship of domains within a larger DSP crystal lattice. A novel Drp1 crystal structure was reported 2 years later by the Daumke lab [51]. Figure 1C highlights similarities in crystal structures of dynamin‐1 (PDB ID: 3SNH) and Drp1 (PDB ID: 4BEJ), as both have G domains in an extended position and adjacent stalks are orthogonal to one another through a dimer interface, primed for assembly. A recently reported dimeric structure of Drp1 (PDB ID: 8T1H, Figure 1C) was determined by cryo‐electron microscopy (cryo‐EM) [37], and the protein density in this solution state exhibited a unique, autoinhibited conformation. When compared with the aforementioned crystal structures of DSPs, the G domains are tucked in, rather than extended, and the relative orientation of the stalks in the dimer pair is aligned in a manner that limits the solvent‐accessible surface area and additional self‐assembly interactions.

X‐ray crystallography has contributed the greatest number of DSP structures to date and was used to determine the structures of dynamin‐1 and Drp1 to high resolution. However, DSPs have traditionally been a difficult target for crystallographic studies because of their inherent self‐assembly properties. This attribute and the presence of several unstructured loops and domains (see below) make it difficult to concentrate the protein to form an ordered lattice without self‐assembly aggregation. Exhaustive studies identified mutations that limit self‐assembly and removed intrinsically disordered regions (IDRs) to facilitate crystallization that preserved the correct folding of core DSP domains [48, 49, 50, 51]. More recently, cryo‐EM has emerged as a powerful tool that allows for structural studies of solution protein multimers and larger assemblies. Refined data collection and direct electron detectors have improved resolution, and enhanced classification methods have allowed scientists to sort structural heterogeneity and flexibility to resolve protein complexes in distinct conformational states [52]. Correspondingly, the past decade has brought unprecedented insight to the assembly properties of DSPs, and ongoing studies will identify conserved features and select differences that confer specialized functions in the cell.

In comparing dimer structures of Drp1 in Figure 1C obtained using different structural methods, we see that crystallization of Drp1 identified an assembly‐primed, active conformation (PDB ID: 4BEJ). As noted above, the core dimer is “open” in a conformation with extended G domains and the stalks splayed, exposing intermolecular interface regions. The cryo‐EM structure of full‐length, wild‐type Drp1 was identified in a more condensed, auto‐inhibited conformation (PDB ID: 8T1H) [37]. The orientation of the G domains in this conformation was tucked in toward the stalk, obscuring highly conserved loops that would otherwise be accessible in solution. In tandem, the intermolecular stalk interface in the dimer complex was aligned in a more parallel conformation, concealing more surface area of the MD and GED when compared to the orthogonal stalk orientation observed in DSP crystal structures. Importantly, this was the first solution structure of Drp1 that included the intrinsically disordered VD. Although there was no apparent density for the VD due to its unstructured nature, its role in regulating assembly was apparent. The flattened dimer interface places VDs from each monomer at opposing ends of the structure. If and when these VDs are engaged by lipid or partner protein interactions, the running hypothesis suggests relief of auto‐inhibitory interactions allows the stalks to sample an open conformation, similar to the crystal structure. Although dynamins contain a structured PH domain for lipid interactions, the sequences connecting this region to the stalk are flexible linkers, allowing PH‐stalk interactions that regulate self‐assembly [23]. In this way, membrane interactions permit assembly of DSP fission machineries at defined membrane sites, where activation invokes conformational changes that promote protein self‐assembly [53]. Additional work is needed to characterize this conformational remodeling within DSP multimers that induce assembly. Remaining gaps in this hypothesis include the determination of pre‐assembly nucleotide binding states and identification of IDR intra‐ and intermolecular interactions as conformational regulators.

## 3 Multimeric Complexes of DSPs in Solution

DSPs, including Drp1 and dynamin, can exist in multiple oligomeric states within the cell. Experiments using size exclusion chromatography paired with multi‐angle light scattering (SEC‐MALS) found that Drp1 is in a dimer/tetramer mixture at physiological salt, and larger multimers, such as hexamers and octamers, are sampled less frequently [7, 54]. Dynamins exist primarily in a tetrameric state, as determined by analytical ultracentrifugation (AUC) [55] and SEC‐MALS [56], though dimers may be present as well [57]. The affinity of dynamins and Drp1 for negatively charged surfaces, including glass slides used in mass photometry or negatively charged lipid‐supported template, highlights the potential interconversion of cytosolic oligomerization states of these proteins [58]. Importantly, dynamin conformation and orientation correlate with enzymatic activity, so this interplay of multimeric states in the context of lipid and nucleotide interactions requires additional investigation.

**FIGURE 2:**
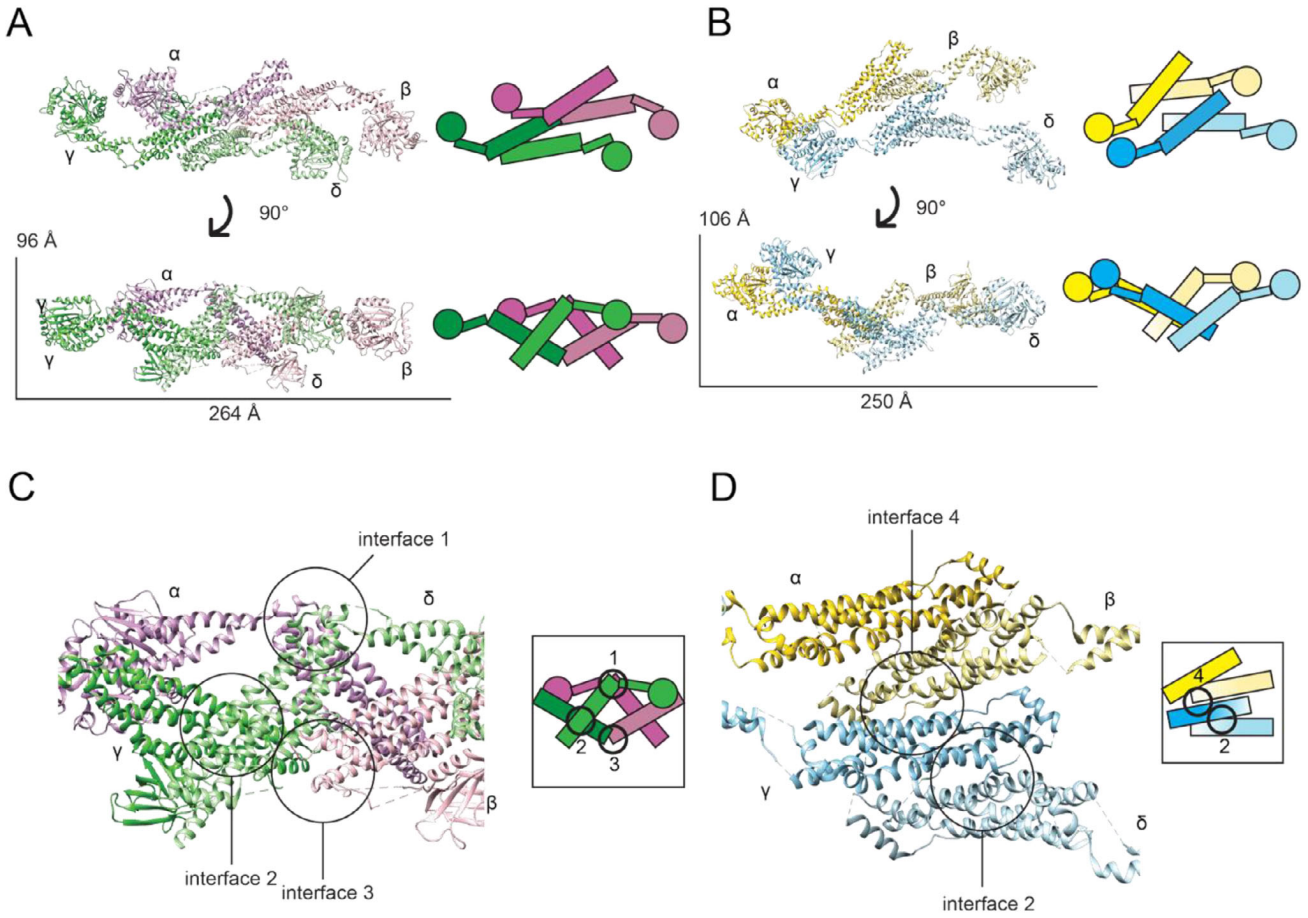
Dynamin superfamily protein (DSP) fission protein oligomer structures. (A) A crystal structure of Dyn3 (PDB ID: 5A3F) was published with an assembly interface formed from two dimers (dimer 1 purple, dimer 2 green). (B) The dynamin‐related protein 1 (Drp1) crystal structure (PBD ID: 4BEJ) was observed to contain an alternative, novel tetramer interface formed from two dimers (dimer 1 yellow, dimer 2 blue). (C) The tetramer interface of Dyn3 is formed through traditional DSP polymer Interfaces 1 and 3, with each dimer pair forming Interface 2. (D) The tetramer interface of Drp1 forms a novel Interface 4, while two dimers were observed to assemble through the traditional Interface 2.

Fundamentally, dynamin and Drp1 dimers represent the core unit of larger multimers, including tetramers [58]. In fact, distinct tetramer structures were determined for dynamin‐3 (PDB ID: 5A3F) and Drp1 (PDB ID: 4BEJ) using x‐ray crystallography (Figure 2A, B) [51, 59]. Both of these complexes are formed via a dimer of two dimers (monomers labeled α and β form one dimer, γ and δ form the other dimer). The individual dimer structures are connected through the highly conserved Interface 2, roughly midway through the stalks of each monomer. Assembly of dimers via Interface 2 is consistent across DSPs, including MxA [48] in humans, cmDnm1 in algae [60], and Mgm1 in fungi [61]. Although relative position of this interface is conserved in DSPs, sequence differences in this region may lead to the heterogeneous sampling of stalk conformations in different DSP dimers, which likely alter self‐assembly properties.

These differences are apparent in the observed interactions within tetramer structures of dynamin and Drp1 (dimer of dimers). In dynamins, Interfaces 1 and 3 are the backbone of tetramer formation (Figure 2C). Interface 1 meets at the proximal stalk‐BSE hinge of α and δ monomers from opposing dimers, whereas Interface 3 meets at the distal end of the stalks of the β and γ monomers. The residues involved in Interface 1 form a hydrophobic network to stabilize this interaction, which is largely conserved in DSPs [59]. Interface 3 is also broad, involving the core hydrophobic interactions of stalk loop L2^S^ and the proximal interactions of loop L1N^S^. Mutations in this region, such as the dynamin‐3 G346D and G397D in loops L1N^S^ and L2^S^, respectively, disrupt higher order oligomer formation [59, 62]. Similar mutations at two conserved arginine residues within L1N^S^ and L2^S^ (Residues 361 and 399 in dynamin) prevent self‐assembly. Thus, Interface 3 plays an essential role in DSP self‐assembly [48, 56].

An alternative mode of assembly was observed in a Drp1 tetramer structure. A novel Interface 4 (Figure 2D) was proposed, leading to G domains stacked on opposing sides of growing helical filaments [51]. However, when key residues of Interface 4 were mutated, there was no effect on sedimentation or lipid‐stimulated GTPase activity [51]. As noted earlier, the VD had to be removed, and a conserved GPRP motif in the L2^S^ loop was mutated in the crystallography construct. These changes did not disrupt the core structure, but likely impacted the intermolecular assembly properties of the protein, based on the now apparent autoinhibitory role of the VD on self‐assembly [37] and role of L2^S^ in Interface 3 [63]. Despite this, imaging and cellular studies demonstrated how mutations at Interface 4 caused irregular tubulation of liposomes and failed to rescue mitochondrial fission in Drp1‐null cells [51]. Although no comparable structures among DSPs exist, the inability of Interface 4 mutations to properly assemble suggests that they serve an important role in the larger mitochondrial fission complex.

Since DSPs exist largely in the cytosol when not assembled at membrane constriction sites, hypotheses of unassembled Drp1 and dynamins acting as regulatory factors or signaling molecules have been explored [64, 65, 66]. At the same time, it has been shown that larger multimers of Drp1 in the cytosol are unable to rescue fission in the absence of smaller dimer species [54]. These transient assembly states are thought to represent a storage form of the protein. Similarly, an Interface 3 patient mutation in dynamin‐2 led to larger multimer formation and showed impaired membrane trafficking [67]. Even with these findings, unanswered questions about the role of “storage state” oligomers remain. Are there differences between multimer interactions with lipids and partner proteins? And do similar mechanisms of conformational regulation exist in DSPs other than Drp1 to restrict assembly interfaces? To date, cryo‐EM studies of DSPs have focused heavily on membrane‐bound protein helices, and crystallographic studies have altered key interfaces to ensure protein solubility. Therefore, additional structures of cytosolic multimers are needed to better understand the regulation of self‐assembly within the membrane‐remodeling machineries in this protein family.

## 4 Helical Assembly of DSPs to Promote Membrane Curvature

In order for DSPs to induce membrane curvature, they must self‐assemble into larger helical structures. The aforementioned “open” dimer is generally believed to form the core unit of larger filaments that wrap around the membrane [54, 68]. These larger assemblies form through shared intermolecular stalk interactions, and GTP hydrolysis induces conformational changes that mediate constriction [34, 69]. Structures of DSPs in lipid‐induced helical assemblies have identified key interfaces required for assembly and subsequent constriction. Still, recent studies suggest that different mechanisms to facilitate membrane remodeling exist within the protein family [70, 71].

Dynamins form collars around endocytic budding vesicles, and upon GTP hydrolysis, constrict to perform fission. The first membrane bound structure of a DSP was of dynamin‐1 and used helical symmetry to resolve domain organization within the assembly [53]. This was followed by successive cryo‐EM structures focusing on isolated segments of the helix, highlighting the inherent flexibility in the polymer (PDB ID: 3ZYS [34]). These structures also examined the impact of nucleotide interactions on the polymer, and conformational differences demonstrated how the dynamin helix could impart strain on the underlying lipid bilayer [34, 72]. Additional innovations in data collection and image processing led to significant advances in cryo‐EM structures of DSPs.

**FIGURE 3:**
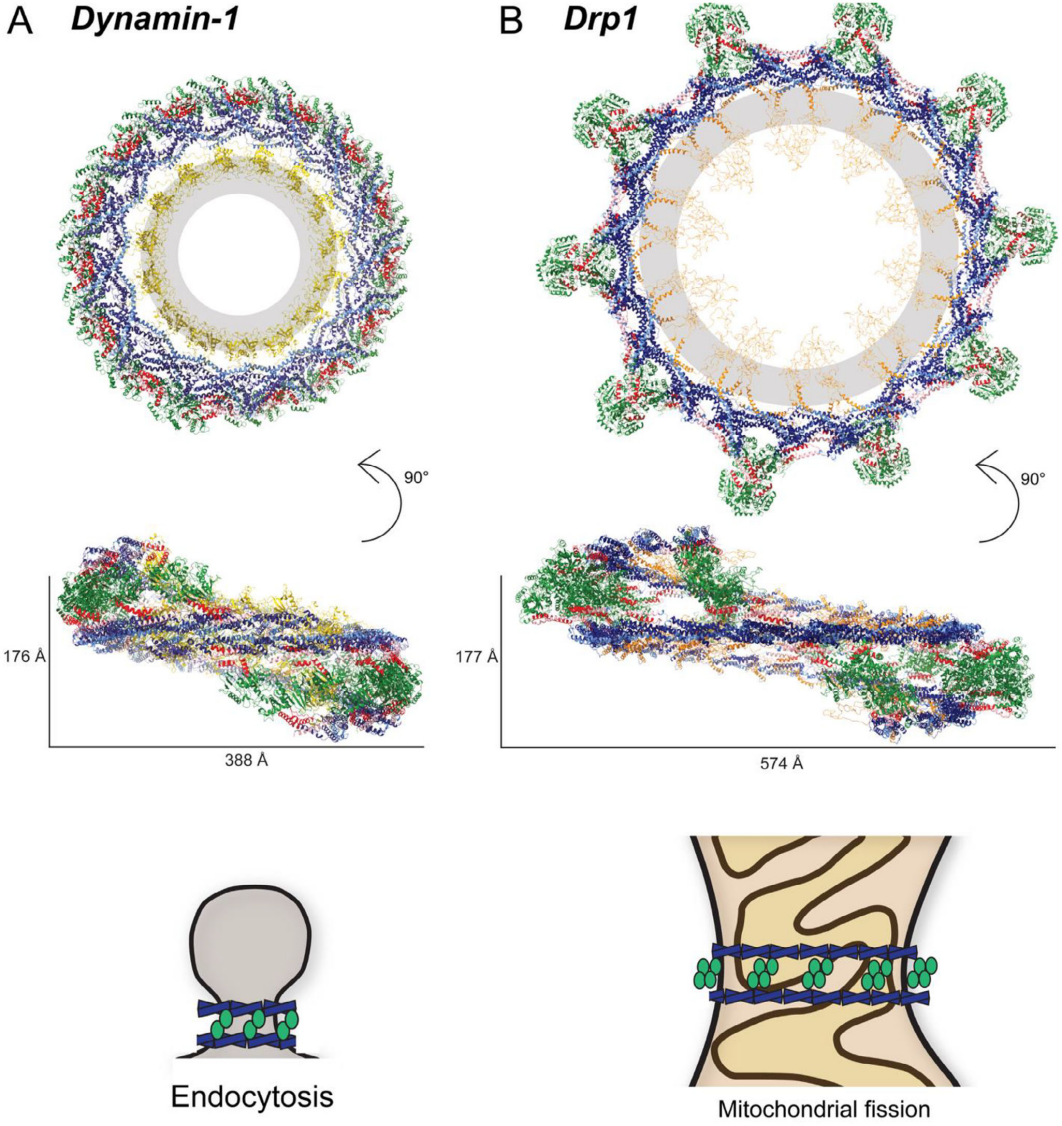
Helical assembly of Dynamin superfamily proteins (DSPs). (A) Dynamin‐1 bound to GMPPCP forms a tight helical assembly with the pleckstrin homology (PH) domain (yellow) projecting into the lipid membrane (gray) (PDB ID: 6DLU). The schematic represents placement of stalks (blue) and G domains (green) in one rung of dynamin assembly. (B) Dynamin‐related protein 1 (Drp1) forms a larger helical assembly with tetrameric G domain (green) interactions protruding from the assembly (adapted from Peng et al.). The schematic represents tetrameric G domain assemblies around the larger diameter of a mitochondrion.

An improved structure of a dynamin helical assembly in a GMPPCP‐bound state was recently resolved to high resolution (Figure 3A), revealing detailed interactions between repeating subunits (PDB ID: 6DLU [73]). This polymer tubulated negatively charged liposomes to form a uniform assembly with an outer radius of ∼40 nm around the membrane. GMPPCP is a non‐hydrolysable nucleotide analog that mimics a GTP‐bound, pre‐hydrolysis state. In this state, the cryo‐EM structure showed 1‐start helices built via three stalk interfaces, numbered 1, 2, and 3 (consistent with cytosolic multimers), starting from the most distal to membrane‐proximal regions. Interface 2 stabilized core, dimer interactions, consistent with previous crystal structures (PDB IDs: 3ZVR [50], 3SNH [49]). Interface 3 formed between the membrane proximal bases of opposing stalks, and these interactions position the PH domain near the lipid bilayer, where they integrate into the outer leaflet. Interface 1 was characterized as having several residues involved in the intermolecular interactions adjacent to the BSE and G domains. Only after multiple mutations were introduced in these interfaces were there disruptions to endocytosis, highlighting the importance of stability from having a broad surface area involved in the interfaces [73].

Formation of the interactions between opposing GTPases (G–G interactions) stabilized inter‐rung interactions within the dynamin helix [68, 73]. Nucleotide binding promotes conformational changes in the ∆PRD construct, and stalk rearrangements generate constriction on lipid‐sensing regions that impinge on the lipid bilayer [39, 73]. These polymers require dynamic interactions wherein adjacent helical turns can transiently dissociate and reassociate to promote membrane remodeling [74, 75], and flexibility in the polymer is apparent in a recent publication comparing the high‐resolution structures of assemblies upon GTP binding and hydrolysis [70]. The Hinshaw lab suggested that dynamin undergoes a two‐step constriction, with GTP binding conferring partial constriction, followed by a hydrolysis‐induced “powerstroke” further constricting around the lipid membrane [34]. Importantly, dynamin‐lipid tubules in a 1‐start GTP‐bound constricted state transition to a 2‐start GDP‐bound super‐constricted state, requiring axial expansion and reorganization of PH domains proximal to the lipid bilayer [70]. The impact of additional interactions with the above‐mentioned SH3‐containing proteins [40] is largely uncharacterized. It is likely that juxtaposed partner–protein interactions influence the dynamin helix since assembly of dynamin‐SH3 complexes stimulates GTPase activity [76] and endophilin was shown to inhibit dynamin‐mediated fission [77]. Moreover, structural studies have largely focused on the local interaction between the PRD of dynamin and SH3 domains [78, 79, 80]. Ongoing investigation of dynamin copolymers with SH3‐containing proteins will define their roles in modulating the contractile machinery.

Fewer studies have explored Drp1 assembly on lipid bilayers, but recent advances in cryo‐EM data analysis methods have highlighted unique Drp1 assembly properties. At the surface of mitochondria, activation of Drp1 dimers from the autoinhibited conformation requires opening of the stalks and extension of the G domains to assemble around the outer mitochondrial membrane [37, 81]. Recruitment of Drp1 can be mediated by partner proteins, including Mff, Fis1, and Mid49/51, as well as patches of cardiolipin (CL) in the membrane [82]. CL is typically found on the inner mitochondrial membrane and can flip to the outer mitochondrial membrane at bordering contact sites under periods of stress or in response to other signaling events [83]. An initial study looked to reconstitute lipid interactions, where Drp1 was assembled on a galactosyl ceramide/phosphatidylserine (GC/PS) nanotube template [84]. However, there was no observable “touch down” of the protein on the lipid, likely owing to the transient nature of the interaction. In contrast, Drp1 contact on the lipid nanotube template was observed when CL was present [84]. This structure provided insight on the role of the VD in promoting CL contacts, but there were unassigned densities in the helical structure after docking Drp1 dimers into the cryo‐EM maps.

Recently, improved structures have been determined for Drp1 bound to negatively charged lipid templates [71]. Pseudoatomic modeling was able to build a lattice structure from repeating asymmetric dimers (Figure 3B). The stalk interactions exhibited conserved Interfaces 1, 2, and 3, largely similar to previous dynamin helical structures, but the positioning of the peripheral G domains was unique [81]. Specifically, the G domains formed a compact tetramer that protruded from the assembly. Based on conformational heterogeneity in the helical samples, one of the G–G pairs in this unique tetramer conformation was found to be transient. Still, the other G–G pair releases and extends along the filaments to maintain inter‐rung interactions that drive constriction of the underlying membrane [81].

Similarities between the assemblies of Drp1 and dynamin can be seen in the asymmetrical extension of individual molecules making up the dimers, as well as the involvement of lipid‐sensing regions (VD [35] and PH domain [39]) wedging into the membrane to generate curvature [73, 81]. Interestingly, Drp1 constriction requires GTP hydrolysis as opposed to GTP binding, which may be a result of the unique interactions in the helical lattice. It was noted that VD–lipid interactions alternated between stronger and weaker membrane integration along the lattice, which may permit the dynamic movement of the protein along the surface of the lipid bilayer following GTP hydrolysis [81]. Given the differences between Drp1 and dynamin ground state helical structures, further research on different nucleotide binding states of assembled DSPs will contribute to our mechanistic understanding of how these membrane fission proteins constrict distinct cellular membranes.

Other DSPs with helical structures include Vps1 [85], MxB [86], and Opa1 [87, 88, 89], and each shows distinctive assembly mechanisms. Although Opa1 tends to form G domain interactions on the exterior of the assembly similar to Drp1, both Vps1 and MxB show more condensed G domain interactions at the radial plane adjacent to stalk interactions, similar to the dynamin‐1 assembly. Importantly, conformational changes that result from nucleotide binding and hydrolysis require a level of flexibility within the protein inter‐ and intramolecular interactions. This flexibility must allow for the G domains to extend and contract, as well as the stalks to form transient interfaces that confer constriction. Accordingly, BSE hinges and loops located in stalks are conserved among DSPs, suggesting a critical role for elasticity in the assembly and subsequent constriction of lipid membranes [90, 91].

## 5 Conformational Rearrangements that Promote Assembly and Constriction

**FIGURE 4:**
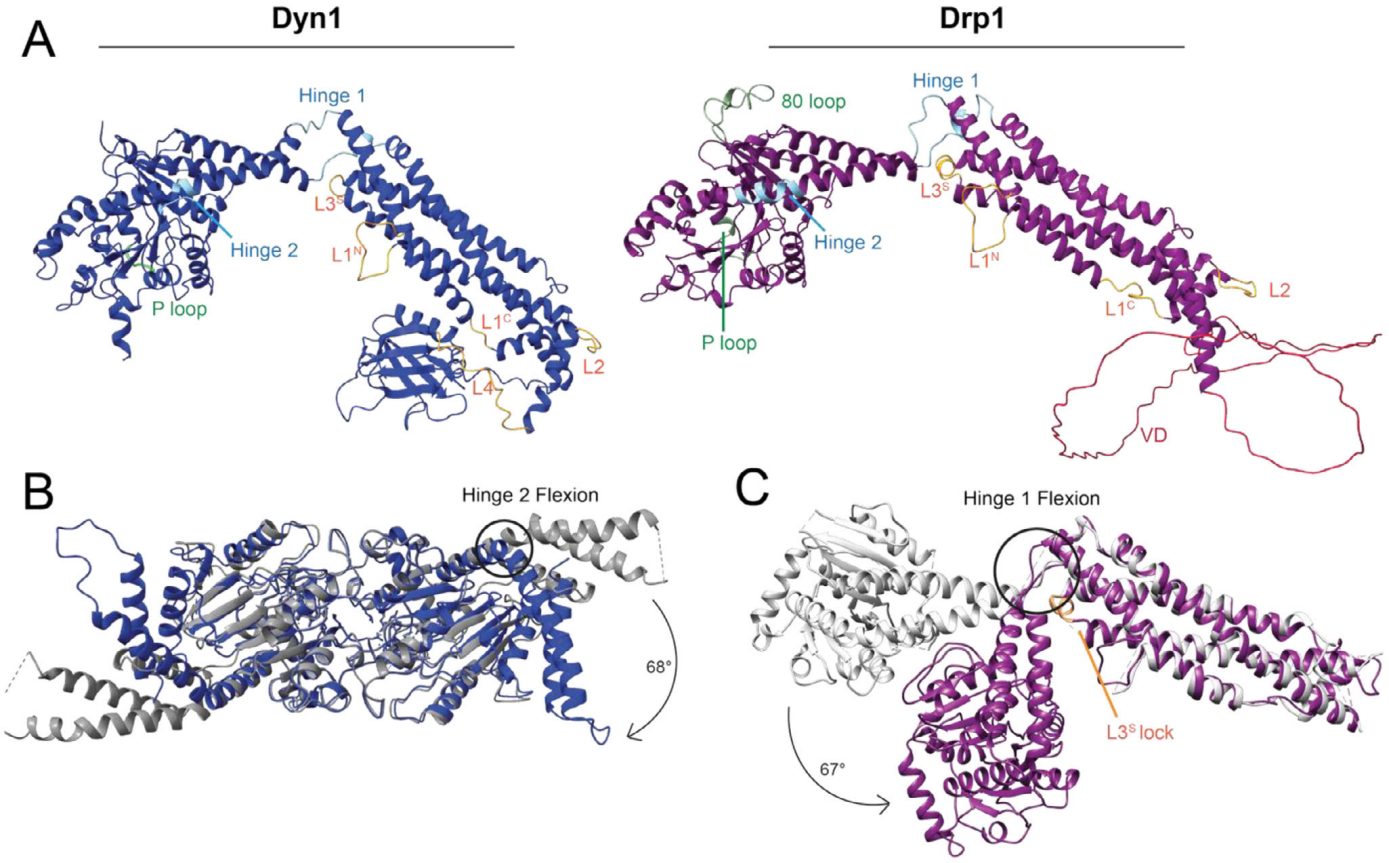
Conserved hinges and loops of dynamin‐related protein 1 (Drp1) and dynamin. (A) Loops and hinges of Dyn1, left, and Drp1, right. AlphaFold structures were used to give representation to disordered regions. (B) A comparison of the bound (PDB ID: 2X2E, in blue) and unbound (PDB ID: 3ZYC, in gray) G domain‐GED (GG) constructs of dynamin. The powerstroke conferred through Hinge 2 results in a 68o change in extension of the bundle signaling element (BSE). (C) A comparison of the crystal structure conformation (PDB ID: 4BEJ, in silver) to the cryoEM structure conformation (PDB ID: 8T1H, in purple). The cryoEM structure represents an autoinhibited state with a 67o compression of the G domain through Hinge 1 against Loop L3^s^.

DSPs have many shared loops and hinges, and mutations at these sites are known to disrupt oligomerization and functional assembly. In Figure 4A, these loops and hinges are labeled on AlphaFold‐predicted structures of Drp1 and dynamin‐1, used to show the full sequence of each protein with IDRs intact [92]. In the G domain, both dynamin‐1 and Drp1 share the aforementioned P loop, which coordinates nucleotide binding via hydrogen bonding with the β‐phosphate of GTP [42]. This loop is conserved in all nucleotidases, and it plays a critical role in coordinating GTP interactions and conformational changes in the helical polymer. Mutations in the P loop are known to prevent coordination of nucleotide phosphates and inhibit hydrolysis [32, 93]. This provides a unique tool to assess the role of GTPase activity on protein structure and function. In dynamin, the K44A mutation does not limit helical constriction, as GTP binding is sufficient to promote conformational remodeling [47], and a post‐hydrolysis, GDP‐bound state led to a super‐constricted lattice [70]. Conversely, Drp1‐mediated constriction requires hydrolysis to mediate constriction, and the homologous K38A mutation is unable to confer conformational changes in response to nucleotide occupancy of the GTP‐binding site [94]. The underlying difference remains unclear, and future studies will inform nucleotide‐induced conformational changes that mediate membrane constriction. It is unclear why this mechanism would be different in distinct DSPs, though the altered inter‐rung interaction between opposing G‐domains may factor into this inconsistency. Additionally, discriminating interactions with specific lipid and partner proteins may be a factor as well.

Another G domain loop, unique to Drp1 and other mitochondrial fission DSPs, is the so‐called 80 loop. This region has been suggested to localize Drp1 to lysosomes and late endosomes in addition to mitochondria in mice [95]. The 80 loop is a 20‐amino acid segment where alternative splicing leads to inclusion or exclusion of Exon 3, which encodes an additional 13 amino acids primarily expressed in brain tissue and skeletal muscle [96]. In enzymatic studies, the inclusion of the extended 80 loop showed a decrease in cooperative G domain activation by inhibiting dimerization and thus decreasing GTPase activity compared to the protein lacking this sequence [36]. In cellular studies, deletion of the entire 80‐loop impaired recruitment to the mitochondria and could not restore mitochondrial fission in a Drp1‐KO cell line [36]. These data suggest that the 80 loop acts to recruit Drp1 to mitochondria and induce G domain oligomerization, while inclusion of Exon 3 in this region adds auto‐inhibitory regulation of GTPase activity required for constriction.

Two hinges have been found to be critical for function in DSPs. Between the G domain and stalk, loops L1^BS^ and L2^BS^ flank the BSE to confer flexibility to permit extension and compaction of the structure. Together, these loops form Hinge 1. In Figure 4C, Hinge 1 of Drp1 is the site of flexion seen when comparing the crystal structure monomer (PDB ID: 4BEJ, in silver) in an active form with the auto‐inhibited monomer observed in the cryo‐EM structure (PDB ID: 8T1H, in purple) [37, 51]. A large, rotational shift of the G domain from its active state to an auto‐inhibited state introduced a novel interaction between the BSE and Loop 3 (L3^S^) of the stalk. This “lock” was investigated using a charge reversal mutation in L3^S^ to disrupt this interaction, and this change yielded a protein with a higher propensity for oligomerization when nucleotide was added [37]. Hinge 2 (previously referred to as the BSE hinge) occurs at a highly conserved proline in helix α2^B^ of the BSE that confers flexibility and induces a powerstroke in dynamin upon GTP hydrolysis [34]. In Figure 4B, nucleotide interactions were examined in G domain constructs lacking the stalk and PH domains. Crystal structures of these dynamin G domain‐GED (GG) constructs were determined in a GTP‐bound state (GMPPCP, PDB ID: 3ZYC) and separately with a transition state analog (GDP‐AlF_4_^−^, PDB ID: 2X2E). There was little change in the dimerization interactions, but a large rotation of the BSE hinge was observed [34, 68]. Beyond the obvious shift of the BSE, a major difference in the G domain of dynamin was the presence of a cation, rather than a water molecule, upon addition of GDP‐AlF_4_^−^. This cation balanced the negative charge of the nucleotide phosphate groups and maintained the dimer interface responsible for cooperative enzymatic activity [34]. Collectively, these hinges are important for dynamin and Drp1 function through their intra‐ and intermolecular interactions that stabilize the inactive state of these DSPs. These hinges also confer critical flexibility needed to accommodate the range of helical geometries that the assembled polymer forms during constriction.

Similarly, loops within the stalks play a variety of functional roles in Drp1 and dynamin. L3^S^ participates in the aforementioned auto‐inhibited state where the G domain and BSE tuck in toward the stalk region, adjacent to the L1N^S^ loop [37]. Conversely, L1N^S^ is critical for DSP self‐assembly, and mutations in this region of Drp1 prevent oligomerization beyond the dimer state [54, 63, 97, 98]. Similar mutations in dynamin and other DSPs limit self‐assembly, and tetramer formation was inhibited by mutations in L1N^S^ as it acts as a binding region for the PH domain of dynamin [48, 59]. L1C^S^ is another loop in the stalk region that was observed in both Drp1 and dynamin structures, but there is limited data available on the role of this loop. It is proximal to the VD and PH domains in Drp1 and dynamin, respectively, highlighting a potential role in regulating self‐assembly. Nearby mutations in L2^S^ also prevent self‐assembly, highlighting its central role in stabilizing Interface 3 [48, 51, 59]. At the base of the stalk, the PH domain connects the MD and GED, while the unstructured VD of Drp1 is in a similar location. Homology between loops in the stalk region of DSPs contributes to conformational elasticity that permits these proteins to assemble around lipid membranes and constrict through alterations in the helical symmetry [70, 99]. Many studies have focused on the loops directly involved in intermolecular interfaces, informing their roles in DSP assembly. The limited understanding of loops not involved in these interactions highlights the need for further investigation into their functional roles, potentially including partner protein or lipid interactions, or protein localization and trafficking.

## 6 Regulation of DSP Assemblies via Sequence Alterations

The carefully orchestrated sequence of events, from assembly to constriction and fission, requires regulation to ensure that membrane remodeling occurs at a defined location in response to cellular cues. In the cases of dysregulated Drp1 or dynamin activity, impaired or excessive membrane fission results in particularly harsh neurodevelopmental and neurodegenerative phenotypes [15, 97]. To sustain cellular health, regulation of DSP activity comes from a variety of sources including gene expression at the level of DNA [100, 101], alternative splicing of mRNA [9, 102], IDRs [36, 41], and numerous post‐translational modifications (PTMs) [103, 104, 105, 106, 107, 108, 109], as well as protein interactions [40, 82, 110] to coordinate trafficking and recruitment. Intermolecular interactions with lipids and proteins have been covered more extensively in other reviews [24, 25]. For this review, specific regulatory factors that alter DSP sequences directly and indirectly through collaborative interactions with partner proteins are discussed.

Following protein translation, Drp1 is regulated by PTMs that include phosphorylation, SUMOylation, O‐GlcNAcylation, acetylation, palmitoylation, ISGylation, and S‐nitrosylation [104, 105, 109, 111, 112, 113]. Notably, the majority of the PTMs target residues in the disordered VD, likely due to enhanced exposure of the primary sequence and lack of globular structure, which permits multiple PTMs to occur at once [114]. These PTMs regulate intra‐ and intermolecular interfaces that impact Drp1 self‐assembly and interactions with partner macromolecules, as well as GTPase activity directly. Some of the more well‐studied PTMs occur at critical serine residues (S616 and S637) in the VD (Isoform 1 numbering) [107, 115]. Phosphorylation by cyclin‐dependent kinase 1 (CDK1) at S616 was initially characterized and found to promote Drp1‐dependent mitochondrial fission in preparation for mitosis [115]. Similar activation by Rho‐associated coiled‐coil kinase 1 (ROCK1) [116] or Ca^2+^/calmodulin‐dependent protein kinase Iα (CaMKIα) [103] results in increased mitochondrial fission under conditions of hyperglycemia and Ca^2+^ dysregulation, respectively. Twenty‐one residues downstream, phosphorylation at S637 by protein kinase A (PKA) [105] results in Drp1 inactivation. Interestingly, a study using phosphomimetics found that negative charges at each site, S616 and S637, led to smaller non‐assembly oligomeric species and decreased GTPase stimulation by partner proteins [108]. These findings suggest that additional factors are involved and indicate crosstalk between these Drp1 phosphorylation sites, highlighting the complexity of PTM regulation [117]. Other PTMs can lead to increased or excessive mitochondrial fission, including SUMOylation of lysine residues in the VD [112, 118], S‐nitrosylation at C644 in neurodegenerative diseases [104, 113], ISGylation at K532 prevents degradation of the protein [111], and O‐GlcNacylation in response to nutrient stress [109, 119]. Collectively, Drp1 PTMs target regulation of the intrinsically disordered VD to control organelle dynamics, but additional research is needed to determine the underlying mechanisms.

Fewer PTMs have been characterized in dynamins. Yet, dynamins were first discovered to be phosphorylated in 1993 by protein kinase C at residue Y597, which led to a 12‐fold increase in enzymatic activity [106]. Conversely, inhibition of dynamin recruitment to membranes by way of phosphorylation at Y774 is important for synaptic growth [120]. S‐nitrosylation of dynamin also regulates endocytosis and apoptotic signals, as cysteines C607 [121] and C86 [122] are targets of modification.

Alternatively, a well‐studied form of regulating dynamin function is through gene expression and splicing. Among the three genes encoding dynamins, there are 25 splice variants that lead to unique patterns of localization and roles in the cell [123]. Dynamin‐1 and dynamin‐3 have partially overlapping roles in synaptic vesicle recycling in neurons, with dynamin‐1 suggested to play a larger role on the presynaptic side and dynamin‐3 on the postsynaptic side [31, 124]. Interestingly, splice variants of dynamin‐1 showed a split in localization at the presynaptic bouton and the cytoplasm, with the former able to perform endocytosis at a much faster rate [125]. Dynamin‐3 splicing results in isoforms that regulate Golgi dynamics or actin‐dependent neuronal remodeling [123, 126]. Dynamin‐2 is involved in clathrin‐mediated endocytosis and splicing yields isoforms targeted to the Golgi in non‐neuronal cells [127]. Collectively, splicing and PTMs confer a variety of functional roles for dynamins within the cell, but underlying mechanisms are largely unknown.

The transcript encoding Drp1, like dynamins, undergoes splicing to create isoforms with alternative functions and expression patterns in distinct tissues. The longest isoforms are typically found in neurons, while shorter isoforms are more ubiquitously expressed [35, 102]. Although each is found primarily in the cytosol, varying rates of enzymatic activity, cooperation with partner proteins, and association with microtubules leads to variance in the ability to rescue mitochondrial fission in Drp1‐null cells [9, 36, 128]. As with dynamins, further research is needed to identify how PTMs and splicing affect the structural characteristics and interfaces discussed previously.

Regulation of DSPs is not limited to primary sequence modifications; in fact, transcription factors, partner proteins, and other external influences affect their functions. Dynamins interact with actin and Bin‐Amphiphysin‐Rvs (BAR) domain proteins, which bind to the disordered PRD through their SH3 domains, for recruitment to membrane surfaces [129]. Again, Drp1 is recruited to mitochondrial sites marked for fission by several partner proteins [82]. At the organellar level, the endoplasmic reticulum also forms contact sites with mitochondria that contribute to Drp1 recruitment [130]. Specifically, dynamins and Drp1 interact with nearby microtubules, as they were initially identified as microtubule‐associated proteins [1, 96]. Collectively, altered interactions between DSPs and key regulator proteins modulate productive assembly of the fission machinery.

## 7 Summary

Recent structural advances have revealed conserved motifs within mammalian fission DSPs and how these features control self‐assembly states. There are many similarities between dynamins and Drp1 in their dimer states, but as they build larger assemblies, the seemingly minor differences in their dimer structures propagate into unique self‐assembly patterns that influence the formation of oligomers that mediate membrane remodeling events. The pre‐assembled tetramer interfaces first observed in dynamin‐3 appear to be conserved in Drp1 assemblies, but unique interfaces in Drp1 solution multimers regulate the formation of larger mitochondrial fission complexes. The IDR of the VD is the most prominent regulatory feature in Drp1, and changes in this region through PTMs and alternative splicing alter protein self‐assembly properties as well as lipid and partner protein interactions. Additionally, the G domain dimerization known to enhance GTPase activity of dynamin assumes a unique organization in the Drp1 helical lattice structure, suggesting distinct conformational rearrangements that promote constriction of underlying lipid templates.

Additional studies will advance our understanding of mammalian fission DSPs and clarify the mechanisms that impart contractile force on defined cellular membranes. Innovative approaches with cryo‐EM and cryo‐ET are poised to observe protein oligomerization and interaction patterns in reconstituted systems and intact cells with minimal interventions to ensure that the assembly properties of native protein are minimally altered. With this foundation in place, additional studies into PTM modifications and targeted sequence changes, including altered splicing or clinically relevant mutations, will be used for comparative studies to define specific shortcomings in self‐assembly or functional binding with partner proteins and lipids. In this way, specific differences will be identified to generate targeted therapeutics that address pathological states driven by DSP dysfunction.

## Author Contributions

**A.N.H**. drafted the initial manuscript and figures, which were subsequently reviewed and edited by **K.J.R**. and **J.A.M**. All authors approved the final manuscript and figures.

## Conflicts of Interest

The authors declare no conflicts of interest.

## Data Availability

Data sharing not applicable to this article as no datasets were generated or analyzed during the current study.
